# Occupational Exposure to Electromagnetic Fields and Health Surveillance according to the European Directive 2013/35/EU

**DOI:** 10.3390/ijerph18041730

**Published:** 2021-02-10

**Authors:** Alberto Modenese, Fabriziomaria Gobba

**Affiliations:** Department of Biomedical, Metabolic and Neural Sciences, University of Modena and Reggio Emilia, 41125 Modena, Italy; alberto.modenese@unimore.it

**Keywords:** electromagnetic fields, occupational exposure, health surveillance

## Abstract

In the European Union, health surveillance (HS) of electromagnetic fields (EMF)-exposed workers is mandatory according to the Directive 2013/35/EU, aimed at the prevention of known direct biophysical effects and indirect EMF’s effects. Long-term effects are not addressed in the Directive as the evidence of a causal relationship is considered inadequate. Objectives of HS are the prevention or early detection of EMF adverse effects, but scant evidence is hitherto available on the specific procedures. A first issue is that no specific laboratory tests or medical investigations have been demonstrated as useful for exposure monitoring and/or prevention of the effects. Another problem is the existence of *workers at particular risk* (WPR), i.e., subjects with specific conditions inducing an increased susceptibility to the EMF-related risk (e.g., workers with active medical devices or other conditions); exposures within the occupational exposure limit values (ELVs) are usually adequately protective against EMF’s effects, but lower exposures can possibly induce a health risk in WPR. Consequently, the HS of EMF-exposed workers according to the EU Directive should be aimed at the early detection and monitoring of the recognized adverse effects, as well as an early identification of WPR for the adoption of adequate preventive measures.

## 1. Introduction

The number of workers occupationally exposed to electromagnetic fields (EMF) is really huge, and there is scant doubt that currently the vast majority of all workers can be regarded as, at least potentially, exposed, with a possibly relevant occupational health risk. According to the U.S. National Institute for Occupational Safety and Health (NIOSH), almost everyone is exposed to the electric and magnetic fields surrounding all electric devices [1]. Considering this, the prevalence of the exposure to electric fields and low frequency magnetic fields in many workplaces can reach almost the 100% of the workers in the modern urban areas [1]. Speaking of static magnetic fields, one of the main occupational sources is that of the magnetic resonance imaging (MRI) scanners; currently, 50,000 active scanners have been estimated worldwide [2]. Applying the proportion of exposed workers per MRI department estimated in the Netherlands [3], it can be assumed that globally more than 2,000,000 workers are exposed to static fields generated by MRI scanners. Finally, considering high frequency fields, more precise data are available; recently, in a large multicenter study involving almost 10,000 subjects and over 35,000 different occupations, a job exposure matrix (JEM) was applied, retrieving exposure data for 468 different occupational groups. The results showed that the 62% of the occupations were exposed to high frequency EMF [4].

According to these premises, as it happens for other occupational risks, the opportunity to develop and implement appropriate health surveillance (HS) programs based on exposure data and risk evaluation has to be considered. Unfortunately, to date, for the medical prevention and early diagnosis of EMF-related effects in exposed workers, no guidelines are available, and to the best of our knowledge, only few indications mainly related to over-exposure situations can be found [5,6]. On the other hand, detailed technical and organizational preventive measures are widely recognized, including individual and workplace protections to be adopted and the risk evaluation practices to be followed [7,8,9,10,11]. For these reasons, we believe that a comprehensive summary on what is known on the medical aspects related to the prevention of occupational EMF exposure risk, according to the definition of the risk provided in the current legislation in Europe, can give an important contribution to the advancement of the research. The possible health risk should be considered not only in relation to the specific scenarios of excessive exposure but also during standard exposure conditions, such those usually reported for the majority of the occupational groups [1,3,4]. Among the main questions that have to be answered by researchers in this field there are: (i) the identification of the workers at risk for adverse effects induced by the occupational EMF exposure; (ii) the possible EMF-related effects to be investigated by the physicians of the exposed subjects, according to the type of EMF; and (iii) the types of medical practices that physicians can follow for the monitoring of the health status of EMF-exposed workers, for the detection of eventual effects associated to the EMF, and for an appropriate medical prevention.

According to the International Labour Organization (ILO) and the International Commission on Occupational Health (ICOH), the methods and objectives of the health surveillance—i.e., the “…procedures and investigations to assess workers’ health in order to detect and identify any abnormality…” [12]—must be clearly defined [13]. These procedures have to be implemented and applied in all the working situations where a relevant occupational risk for the health of the exposed workers exists [12,13]. As mentioned, EMFs are an almost ubiquitous well recognized work-related risk factor [7,8,9,10,11], so in principle, the HS of exposed workers is needed. Nevertheless, a shared definition of the levels above which EMF-exposure can be considered of relevancy for the health of the workers presents some possible issues. In Europe, a specific directive—the “2013/35/EU of the European Parliament and of the Council of 26 June 2013 on the minimum health and safety requirements regarding the exposure of workers to the risks arising from physical agents (electromagnetic fields)” [14], currently adopted in a large number of the European member states—requires an EMF-related risk assessment in workplaces. Based on the results of this evaluation, also considering specific occupational limit values provided as reference, companies are required to evaluate the need of implementing adequate preventive measures, including the HS of workers at risk [7,14].

The objective of this article was to define the main criteria to be considered for the implementation of an appropriate HS of EMF exposed workers, including indications on the relevant exposure levels, on the details of the HS, and on exposure limitations eventually needed for an adequate prevention of EMF-related effects. All these aspects will be discussed, while also considering the possible presence of workers with particular individual conditions determined to have an increased susceptibility to EMF-related risks.

## 2. General Considerations on Occupational Electromagnetic Fields Exposures Relevant for the Health Surveillance of the Workers

Several occupational sources can be relevant when considering the issue of the occupational exposure to EMF. Different types of EMF, corresponding to specific regions of the spectrum, can have specific interaction mechanisms and effects in biological tissue. For the purposes of this manuscript, we used a simplified EMF classification (Table 1); we discuss the main health problems related to the exposure of workers to static magnetic fields (SMF), extremely low frequency magnetic fields (ELF-MF), intermediate frequency EMF (IF-EMF), and high frequency EMF (HF-EMF, including radiofrequency (RF) microwaves, and millimeter and terahertz waves) (Table 1) [7,8,9,10,11].

According to the previously mentioned Directive 2013/35/EU, employers are required to “assess all risks for workers arising from electromagnetic fields at the workplace” [14]. Several sources and databases, useful to identify potentially relevant EMF occupational exposures, are available. Considering ELF-MF, the main examples include the job exposure matrix (JEM) promoted by the NIOSH, based on the results of a personal exposure assessment in 2317 workers [15], or the JEM proposed in Italy based on two days of personal monitoring of ELF-MF exposure in 543 workers engaged in various industrial sectors [16]. Other databases including measurements for the most common sources of EMF exposure in the workplaces and covering different EMF frequencies are the one proposed by Vila et al. [17] and the archive freely available through the Italian website *Physical Agents Portal* [18]. Finally, a mention is deserved in the case of magnetic resonance imaging (MRI) operators, i.e., workers with possibly relevant exposure levels to SMF and time-varying EMF, for whom various measurements have been performed and the results published [19].

In order to give some practical indications to the companies for the application of the European Directive, a specific “non-binding guide” has also been published by the European Commission [7]. This guide reports, among other things, a list of occupational activities with EMF sources potentially requiring a specific risk assessment. In addition to the types of sources and activities, the risk evaluation process and the protections to be adopted have also to consider the possible presence in the company of workers at particular risk, such as those with active medical devices, who are more susceptible to EMF effects even in cases of quite low exposure (as is further discussed in the next sections of the manuscript). In these situations, outlined in a non-exhaustive manner in Table 2, based on the results of a risk evaluation, an appropriate health surveillance should be considered. Moreover, it should be noted that the results of the risk assessment, and consequently, the preventive measures taken—including an adequate information and training of the workers, the need of a health surveillance, and the information on the conditions determining a particular risk—have always to be communicated to the workers by the employer or its representatives [7,14]. As for other occupational risks, the protective and preventive interventions applied can be collective and individual measures, including when possible the elimination of the hazardous EMF source, or its substitution with a less hazardous one, and then other technical (e.g., shielding of the EMF) and organizational (e.g., delimitation and restriction of access, safety signs and notices) measures. Finally, when the previous collective protections are not sufficient to limit the risk, personal protective equipment such as special shoes, work suits, gloves, and eyewear can be applied based on the specific type of EMF inducing the occupational exposure [7,14].

## 3. An Overview of the Adverse Effects Related to Electromagnetic Fields Exposure of Relevancy for the Health Surveillance of the Exposed Workers according to the Directive 2013/35/EU

As clearly explicated in the preamble, the Directive 2013/35/EU has the objective to prevent “all known direct biophysical effects and indirect effects caused by electromagnetic fields”, while the suggested long-term effects of EMF are not addressed as “there is currently no well-established scientific evidence of a causal relationship” [14]. Nevertheless, in the Directive it is also explicitly taken into account that in cases in which “…well-established scientific evidence emerges, the Commission should consider the most appropriate means for addressing such effects” [14]. Furthermore, when transposing Directive 2013/35/EU, each Member State may decide to adopt more restrictive requirements. As a result, even if the general frame is the same, some differences among the European member states in the approach to the prevention of occupational EMF risk, and consequently also to HS, are possible. 

Direct biophysical effects are defined as “effects in the human body directly caused by its presence in an electromagnetic field” [14]. They can be thermal effects in cases of exposure to high frequency fields, inducing an increase of the temperature in the biological tissues. The other direct effects are the non-thermal ones, related to an induction of currents as a consequence of SMF (including movements in the SMF) or of ELF-MF that can stimulate muscles, nerves, or sensory organs, with possible temporary annoyance or detrimental effects involving different nervous or muscle functions (including brain and cardiac functions) (Table 3). Intermediate frequencies can induce both types of direct effects [7,8,9,10,11,20]. Direct biophysical effects are further classified into sensory effects and health effects [7,8,9,10,11,20]. Sensory effects can be considered somewhat minor adverse effects, as their main characteristics are that they are transient and reversible, with no major consequences on the health status of the workers, if not associated with work accidents. These effects are mainly related to relevant SMF and ELF-MF exposure, include vertigo, nausea, perception of a metallic taste in the mouth, magnetophosphenes, minor changes in brain function, tingling sensation due to the stimulation of the nerves, and others [9,10]. On the other hand, health effects are more severe effects, possibly resulting in an impairment of the health of the worker. These effects are, in general, induced only in cases of high exposure levels to EMF, and they can be related to both non-thermal and thermal mechanisms. Non-thermal health effects are, e.g., alterations of the blood flow in the limbs and alterations in the brain and heart functions related to intense SMF exposure, or, e.g., pain due to the stimulation of the nerves, involuntary contractions of the muscles, and alterations of the cardiac rhythm in case of high exposure levels to ELF-MF. Thermal health effects are due to an excessive temperature increase of the biological tissue, possibly resulting in thermal burns involving the whole body or specific body districts (e.g., thermal eye damage with cataracts or skin burns) after intense exposure to high frequency fields. Additionally, in these cases, intermediate frequencies can induce both types of effects [5,6,7,8,9,10,11,20].

Indirect effects are defined as “effects caused by the presence of an object in an electromagnetic field, which may become the cause of a safety or health hazard” [14]. Among the other indirect effects, electromagnetic interference is one of the main issues, being potentially responsible for the malfunctioning of various medical electronic devices, e.g., cardiac pacemakers, implantable cardioverter-defibrillators (ICD), insulin pumps, or other implanted or body-worn active medical devices (Table 3). Other indirect effects are contact currents, determined by the induction of an electric current in the body through a contact or close proximity with a charged object/surface, inducing a range of effects from a simple perception of electrostatic charging or spark discharges to hocks, involuntary muscle contractions, or burns. The contact currents are mainly caused by SMF, ELF-MF, or IF-EMF, but possibly also by RF. Another indirect effect of potential interest is the attraction of ferromagnetic objects within a SMF, possibly inducing the so called “projectile effect”, which can cause severe injuries [7,8,9,10,11,20] (Table 3). Other indirect effects, such as the initiation of electro-explosive devices, fires, and explosions are also possible, even if less interesting from a medical-prevention point of view (Table 3).

## 4. Occupational Exposure Limits according to the EU Directive and Prevention of the Possible EMF-Related Adverse Effects

The Directive 2013/35/EU defines specific exposure limit values (ELVs) and action levels (ALs) for the prevention of the previously mentioned biophysical direct effects and of the indirect effects (even if, in this latter case—as it is further discussed in the next section—it cannot be fully ensured that respecting the limit values can prevent all the indirect effects, especially in the case of the electromagnetic interference risk with active medical devices) [2,7]. These exposure limits and action levels are different compared to other occupational physical risk factors—e.g., the occupational limit values and the action levels available for noise or vibration exposures—and they have precise definitions and meanings, mainly derived by the guidelines of the International Commission on Non-Ionizing Radiation Protection [8,9,10], recently updated for the high frequency fields [11]. The definition of ELVs is explicitly reported in the text of the European directive: ELVs are “established on the basis of biophysical and biological considerations, in particular on the basis of scientifically well-established short-term and acute direct effects, i.e., thermal effects and electrical stimulation of tissues” [14]. Two types of ELVs are defined in the directive: sensory effects ELVs, i.e., “ELVs above which workers might be subject to transient disturbed sensory perceptions and minor changes in brain functions”, and health effects ELVs, i.e., “those ELVs above which workers might be subject to adverse health effects, such as thermal heating or stimulation of nerve and muscle tissue” [14]. Different in comparison to other occupational risks, in cases of EMF with respect to ELVs, the directive almost ensures the absence of any adverse direct biophysical effect in healthy subjects, while not in workers at particular risk. In general, EMF exposures exceeding the ELVs are necessary to induce direct effects (e.g., the induction of nerve stimulation and involuntary muscle contraction), but only at levels significantly above the ELVs is it possible to observe direct health effects, such as changes in blood flow and/or in heart rate [5,6,7,8,9,10,11,20]. Considering the importance of respecting the ELVs for the prevention of direct effects related to EMF exposure, but on the other hand considering also the operational difficulties in evaluating individual EMF exposure warranting the compliance with the ELVs (assessable by numerical simulations only), the European Directive also introduced action levels (ALs), which are “operational levels established for the purpose of simplifying the process of demonstrating the compliance with relevant ELVs or, where appropriate, to take relevant protection or prevention measures”. Based on the respective ELVs, two types of ALs are defined: low ALs and high ALs, which, for magnetic fields, are respectively the “levels which relate to the sensory effects ELVs” and “to the health effects ELVs” [14]. Accordingly, a risk assessment process evaluating the exposure level in a specific workplace below the low ALs also ensures the respect of the sensory effects ELVs, and, consequently, in that workplace no sensory effects can be expected for the exposed workers, and further preventive measures, such as the health surveillance, should be considered only in cases of the presence of workers at particular risk.

## 5. Workers at Particular Risk for Electromagnetic Fields Exposure

The Directive 2013/35/EU mentions workers at particular risk for the first time in its fourth article, stating that employers have to give particular attention in the risk evaluation process to the prevention of any effects on the health and safety of workers at particular risk [14]. There is not an explicit definition of the term “workers at particular risk” in the European directive, but in an analogy with other work-related physical, biological, and chemical risks, these workers can be considered as subjects with conditions possibly determining an increased susceptibility to the health risk related to EMF exposure. Unfortunately, no exhaustive list of these conditions has been currently defined: the European directive only mentions “workers who have declared the use of active or passive implanted medical devices, such as cardiac pacemakers, or the use of medical devices worn on the body, such as insulin pumps” and “pregnant workers who have informed their employer of their condition” [14]. Workers with active implanted medical devices (AIMD) may be at an increased risk of interference of EMF with the devices; among the main examples of these devices are cardiac pacemakers, cardiac defibrillators, cochlear implants, brainstem implants, inner ear prostheses, neurostimulators, retinal encoders, implanted drug infusion pumps, and others [7,21]. EMF interference with AIMDs can be possible also in the case of relatively low EMF exposure, possibly below the 2013/35/EU directive ELVs, causing temporary disturbances of the sensing and/or stimulating functions of the devices, or, in the worst cases, determining permanent malfunctions, deactivations needing resets of the implants, and even inappropriate or unneeded stimulations [22,23,24,25,26]. Of course, interference problems can be possible also in case of body-worn medical devices, such as external hormone infusion pumps, hearing aids, continuous glucose monitoring systems, and metalized drug-delivery patches [7,21]. Fortunately, considering the current scientific literature, published reports of in vivo malfunctions of AIMD or of active body-worn devices in EMF-exposed workers are rare, possibly suggesting that in cases of quite low EMF exposure levels, as usually happens in the majority of the workplaces, no relevant interferences appear [27]. In cases of the appearance of interference, usually only older devices—e.g., in the case of pacemakers those with unipolar configurations, and only in case of quite relevant exposure levels, e.g., determined by close proximity with a welding cable or with an electronic article surveillance gate—can be affected [28]. Nevertheless, in cases of quite high EMF exposure levels, as e.g., in the case of operators working close to a MRI scanner, newer devices can also have interference problems, and this is the reason why even patients with an MRI-conditional AIMD need an appropriate setting for their devices when undergoing a procedure [29,30].

Furthermore, workers with implanted non-active devices, in cases of a presence of metallic parts (a condition that could also happen in old AIMDs), are considered at particular risk for EMF-exposure. A non-exhaustive list of these devices includes: artificial joints, pins, plates, screws, surgical clips, aneurism clips, stents, prostheses of various types (heart valve, orthopedic, eye/retinal, etc.), annuloplasty rings, metallic contraceptive implants, and others [7,21,31]. The metallic parts of these devices can interact with the EMFs resulting in indirect effects such as mechanical ones, possibly causing the dislocation of the device (mainly in case of high SMF exposure) or the induction of currents determining the heating of the device and an inflammatory reaction of the body tissue in contact with the equipment [31,32]. Nevertheless, different in comparison with the interference with AIMDs, these indirect effects involving passive devices are of concern only in cases of quite high EMF exposure levels for subjects with devices containing a quantity of metal sufficient to interact with the fields, resulting in an appreciable effect [7,8,9,10,11]. It should be mentioned here that also nonmedical (and non-active) body inclusions are possible, and they may represent a risk in case of metallic components, e.g., splinters, body piercings, pigments used in tattoos, and others.

Finally, according to Directive 2013/35/EU, another condition potentially inducing a particular risk for EMF-exposed workers is pregnancy. In this case, the possibility of direct effects must be considered, as some published data suggest an increased susceptibility of the fetus to thermal effects related to HF-EMF [33], while no specific adverse outcomes seem associated with SMF and ELF-EMF exposure [8,9,10,11,20]. Recently, some quite large epidemiological studies on cohorts of pregnant women with ELF-MF exposure [34,35] and with exposure to higher EMF frequencies from other sources [36,37] have been published; no associations with adverse maternal outcomes were found for ELF-MF, while possible associations with outcomes as preterm births were reported for higher frequencies, and the results deserve further confirmation in future studies.

## 6. Indications for the Health Surveillance of Workers Exposed to Electromagnetic Fields

According to the Directive 2013/35/EU, HS is aimed at the prevention and early diagnosis of direct biophysical effects and indirect effects related to occupational EMF exposure, also considering the possible presence of workers at particular risk [14]. HS, as happens also in cases of other occupational risks, can include medical examinations, biological monitoring, questionnaire administration to record relevant health conditions, symptoms or effects, and other examinations [12]. Nevertheless, it should be noted that, according to the ICOH international code of ethics for occupational health professionals, biological tests, as well as other investigations, have to be chosen for their validity and relevance for the protection of the workers’ heath, especially considering their sensitivity, specificity, and predictive values, avoiding the use of measures that are not reliable or that are insufficiently predictive screening tests or investigations [13]. In the case of occupational EMF exposure, it has to be noted that to date, no available specific laboratory tests or other medical investigations have been demonstrated as useful for the prevention and early detection of indirect and direct biophysical EMF-related effects. Even in the special cases of HS needed according to the EU directive, when workers report any undesired or unexpected health effect possibly related to EMF-exposure and/or when exposures above the ELVs are detected [14], no standard indications on the possible valid content of such HS are currently available. This is in line with the above-reported observations suggesting that no increased occurrence of adverse health effects should be necessarily expected in cases of overexposure to EMF, while the reporting of transient and rapidly reversible subjective symptoms, possibly related to sensory effects, can be plausible, especially in cases of exposure to strong SMF in MRI activities [5,6,38,39,40,41,42,43]. Nevertheless, also in these special cases of HS, the main issues may be posed in cases of the presence of workers with conditions of particular susceptibility to the EMF-risk, such as those with AIMDs.

Accordingly, the general indications for the HS of EMF-exposed workers are the same as in cases of routine HS or of extraordinary HS (NB: extraordinary HS is needed, according to the European legislation, in cases of overexposure situations or of reporting of effects), requiring an appropriate in-depth medical examination of the worker provided by an occupational physician adequately trained in the prevention of EMF-related risks, with detailed investigation of the possible conditions of particular susceptibility and of possible EMF-related symptoms, considering the use of ad hoc questionnaires to facilitate the anamnesis [7,38,39,40,41,42,43,44]. Specific medical consultations, laboratory tests, diagnostic exams, and other medical examinations need to be decided case by case based on the specific occupational activity, on the type of EMF, on the exposure level, and on the suspected effect to be further investigated. A specific mention is deserved here for the HS of MRI operators, for whom an increased reporting of several subjective symptoms has been documented in several publications [38,39,40,41,42,43]. These symptoms include some nonspecific clinical signs, such as migraine, asthenia, and memory loss, which can be associated also with other occupational and non-occupational risk factors, including high job stress levels [38,39,40,41,42,43]; but recently, a group of five more specific symptoms has been proposed [40]. These five symptoms are vertigo, nausea, head ringing, magnetophosphenes, and the perception of metallic taste [38], and they can be possibly focused and monitored for their evolution during the HS of MRI operators and possibly of other workers with relevant exposures to SMF and ELF-MF, as they can be related to a direct sensory effect, based on the induction of currents in the body. An investigation of these symptoms can be important during HS activities, at least of MRI operators; occupational physicians should periodically monitor the possible causes of these symptoms, changes in frequency/severity, the need for drug therapies, and associations with a particular work organization in order to identify appropriate preventive initiatives, and in particular, adequate information and training (e.g., on the need to avoid rapid movements close to an MRI scanner) of the workers.

Anyway, as in the case of the EMF workplace risk assessment, HS is also probably among the most delicate issues there is when it comes to the prevention of effects involving workers at particular risk and the adequate protection of their health and safety, especially considering those with AIMDs, who are, as reported in the previous sections, at risk for interference effects, possibly severe, also in cases of quite low EMF exposures, even exposures significantly below the ELVs. Accordingly, identification of the conditions determining such particular risk for workers is for sure one of the most important activities during the HS of EMF-exposed workers [44]. All the conditions possibly determining an increased risk of indirect effects due to the presence of AIMDs, implanted passive devices, and body-worn active or passive devices should be checked and exhaustive information on these conditions has to be given to the workers: as the conditions are several, the use of precompiled tools, such as the questionnaire usually administered to the patients before performing a diagnostic MRI examination, can be useful. As it can be deduced according to the Table 2, a specific problem that may be posed is the case of the presence of workers at particular risk, and especially those with AIMDs in workplaces where an EMF risk would not be expected for other workers without conditions of particular susceptibility, e.g., in an office and where it is possible that a specific HS program is not activated. In these cases, considering that the employer may be not aware of the presence of such particular conditions, it is highly important to have detailed information on the workers, with the help of signals indicating the sources possibly inducing interference problems with AIMDs and also reporting the required distances to be respected. Having done this, in case a worker reports the presence of a particular risk, or of a more general possible problem with EMF exposure, this can be reason for an occupational physician to perform a medical examination within an HS program, identifying the eventual problems or conditions of particular susceptibility and defining the need of taking adequate preventive measures. Obviously, the need for specific information and training of the workers is required only in cases of possibly relevant occupational EMF-exposure levels. Information on the conditions of particular risk are one of the most important aspects to be explored in detail during training activities, and such information has to be periodically repeated to the workers.

As a final note on the indications of the HS of EMF-exposed workers, it should be remembered that, according to the EU directive, the collective results of the HS have to be adequately collected and preserved [14], as they represent an important source on which to build more scientific knowledge on possible EMF-related effects and on the conditions of particular susceptibility to the risk.

## 7. Conclusions

The occupational exposure to EMF is a recognized and diffused occupational risk factor, potentially involving a very huge number of workers. Accordingly, as for other occupational risks, the opportunity to implement an appropriate HS of these workers must be considered. As we have seen, in European Union, HS of EMF-exposed workers is mandatory according to a specific directive (2013/35/EU). This directive is aimed at the prevention of known direct biophysical effects caused by electromagnetic fields—such as the stimulation of muscles, nerves or sensory organs, and limb currents—and thermal effects, as well as of the indirect effects, while long-term effects are not addressed as scientific evidence of a causal relationship is considered inadequate. The objectives of HS are clear: the prevention and early detection of EMF’s adverse effects, in the case of both usual working exposure conditions and overexposure (i.e., accidental exposures or extraordinary situations where an exceeding of the occupational limit values are allowed), but to date there is still scant available data on the specific details of the HS. Further research is needed to evaluate the effectiveness of HS programs for the prevention of EMF risk in workers. A first issue is that no specific laboratory tests or other medical investigations have been demonstrated as useful for the prevention and early detection of the abovementioned EMF-related adverse effects; these points still represent important open questions that we hope can be solved soon, based on upcoming scientific evidence. Another problem is the existence of the workers at particular risk, i.e., subjects with specific pathological or physiological conditions possibly inducing an increased susceptibility to the EMF-related risk, as is the case of active implanted medical devices (but other pathological or also physiological conditions, such as pregnancy, are also possible, and currently no recognized exhaustive list of these conditions is available according to the scientific literature). An exposure below the proposed occupational exposure limit values (ELVs) is usually adequately protective against the direct and indirect EMFs effects, but in these workers, relatively low exposure, possibly similar to the levels of the general population, can induce a health risk. Consequently, the main goals of a HS of EMFs-exposed workers according to the EU directive should be the early detection and monitoring of any of the described adverse effects, as well as the early identification of the workers at particular risk. Providing adequate information to workers about the risk, the exposure levels, and the EMF-related effects, including the conditions possibly inducing a particular susceptibility, is also a fundamental part of appropriate prevention for EMF-exposed workers. Finally, the collective results of the HS should be adequately collected and preserved, as they may represent an important source with which to further develop scientific knowledge of possible EMF-related effects, on the conditions of particular susceptibility to the risk, and, eventually, on the possible thresholds.

## Figures and Tables

**Table 1:** Simplified classification of electromagnetic fields relevant for the evaluation of the occupational exposure risk.

Classification	Sub-Class/Division	Frequency
Static fields	Static electric fields	0 Hertz
	Static magnetic fields	0 Hertz
Low frequencies fields	Extremely low frequency electric fields	1–<300 Hertz
	Extremely low frequency magnetic fields	1–<300 Hertz
	Intermediate frequency electromagnetic fields	300 Hertz–<100 kilohertz
High frequencies fields	Radiofrequencies electromagnetic fields (or radio waves)	100 kilohertz–<300 megahertz
	Microwaves	300 megahertz–<30 gigahertz
	Millimeter waves	30–<300 gigahertz
	Terahertz waves	300 gigahertz–10/30 terahertz

**Table 2:** Indications for electromagnetic fields (EMF) risk assessment in different occupational settings, summarized and adapted from the “non-binding guide” of the European Commission [2]: non-exhaustive list.

Type of Equipment or Workplace		Risk Assessment Required in Cases of:		
		Absence of Workers at Particular Risk	Presence of Workers at Particular Risk	
			Without Active Implants	With Active Implants
• Wireless communications				
Use of phones, cordless, mobiles		NO	NO	YES
Workplaces containing phones, cordless, mobiles		NO	NO	NO
• Office				
Audio-visual equipment with radiofrequency transmitters		NO	NO	YES
Office equipment (e.g., photocopiers, paper shredders, etc.)		NO	NO	NO
• Infrastructure (buildings and grounds)				
Base station antennas	inside operator’s designated exclusion zone	YES	YES	YES
	outside operator’s designated exclusion zone	NO	NO	NO
Use of garden appliances (electric operated)		NO	NO	YES
Workplaces containing electric garden appliances		NO	NO	NO
• Electrical supply				
Generators and emergency generators—work on		NO	NO	YES
inverters, including those on photovoltaic systems		NO	NO	YES
• Light industry				
Manual arc welding processes *		NO	NO	YES
Dielectric heating and welding		YES	YES	YES
Industrial magnetizer/demagnetizers		YES	YES	YES
• Heavy industry				
Industrial electrolysis, furnaces, arc melting		YES	YES	YES
• Construction				
Work in close proximity to, e.g., concrete mixers, vibrators, etc.		NO	NO	YES
Microwave drying, in construction industry		YES	YES	YES
• Medical equipment for diagnosis or treatment				
Not employing EMF for diagnosis or treatment		NO	NO	NO
Using EMF (e.g., magnetic resonance imaging, short wave diathermy, transcranial magnetic stimulation)		YES	YES	YES
• Transport				
Radar, air traffic control, military, weather, and long range		YES	YES	YES
Trains and trams, electrically driven		YES	YES	YES

* following good practice and not supporting cable on body.

**Table 3:** Main direct and indirect effects related to electromagnetic fields exposure considered in the Directive 2013/35/EU.

Electromagnetic Fields Frequency Involved	Direct Biophysical Effects	Indirect Effects *
Low frequency	Non-thermal effects (stimulation of muscles, nerves, or sensory organs, inducing temporary annoyance or leading to a possible detrimental effect, e.g., affect cognition or other brain or muscle functions, or inducing safety risks)	Interference (with medical electronic equipment and devices, including cardiac pacemakers and other implants) Contact currents Other indirect effects (as the “projectile risk” in static fields, the initiation of electro-explosive devices and fires and explosions)
Intermediate frequency	Both of thermal and non-thermal types of effects are possible	
High frequency	Thermal effects (determining an increase of the temperature in biological tissue)	

* NB: with the exception of the “projectile risk”, which is a consequence of the magnetic attraction of metallic objects placed in static fields, the indirect effects can be related to all the electromagnetic field frequencies (low, intermediate, high).
